# Nrf2 protects against myocardial ischemia-reperfusion injury in diabetic rats by inhibiting Drp1-mediated mitochondrial fission

**DOI:** 10.1515/med-2023-0711

**Published:** 2023-06-16

**Authors:** Xiao-Li Wang, Qian-Qian Zhu, Alimujiang Simayi, Gui-Ping Xu

**Affiliations:** Department of Anesthesiology, People’s Hospital of Xinjiang Uygur Autonomous Region, Xinjiang Clinical Research Center for Anesthesia Management, Urumqi 830001, China

**Keywords:** diabetes mellitus, myocardial ischemia-reperfusion injury, Nuclear factor E2-related factor 2, dynamin-related protein 1, dimethyl fumarate

## Abstract

Mitochondrial dysfunction and oxidative stress are considered to be two main drivers of diabetic myocardial ischemia-reperfusion injury (DM + MIRI). Nuclear factor-erythroid 2-related factor 2 (Nrf2) and Dynamin-related protein 1 (Drp1) play central roles in maintaining mitochondrial homeostasis and regulating oxidative stress, but the effects of the Nrf2-Drp1 pathway on DM-MIRI have not been reported. The aim of this study is to investigate the role of the Nrf2-Drp1 pathway in DM + MIRI rats. A rat model of DM + MIRI and H9c2 cardiomyocyte injury were constructed. The therapeutic effect of Nrf2 was assessed by detecting myocardial infarct size, mitochondrial structure, levels of myocardial injury markers and oxidative stress, apoptosis, and Drp1 expression. The results showed that DM + MIRI rats had increased myocardial infarct size and Drp1 expression in myocardial tissue, accompanied by increased mitochondrial fission and oxidative stress. Interestingly, Nrf2 agonist dimethyl fumarate (DMF) could significantly improve cardiac function, mitochondrial fission, and decrease oxidative stress levels and Drp1 expression after ischemia. However, these effects of DMF would be largely counteracted by the Nrf2 inhibitor ML385. Additionally, Nrf2 overexpression significantly suppressed Drp1 expression, apoptosis, and oxidative stress levels in H9c2 cells. Nrf2 attenuates myocardial ischemia-reperfusion injury in DM rats by reducing Drp1-mediated mitochondrial fission and oxidative stress.

## 1 Introduction

Diabetes mellitus (DM) is a chronic disease that seriously threatens human health, and cardiovascular disease is the main cause of death in DM patients [1]. Although treatment strategies have been significantly improved, DM patients are still susceptible to cardiomyopathy [2]. Myocardial ischemia/reperfusion injury (MIRI) is a key feature of increased susceptibility to diabetic cardiomyopathy [3,4]. DM animals and patients are mostly resistant to ischemic preconditioning and drugs to protect the heart from ischemia/reperfusion (I/R) injury in non-DM subjects [5,6]. These data suggest that the loss of endogenous protective mechanisms caused by hyperglycemia or DM itself may contribute to the poor prognosis of DM subjects with MIRI [7,8]. Hyperglycemia causes excessive reactive oxygen species (ROS) production, resulting in enhanced oxidative stress and is thus responsible for aggravating MIRI in DM patients. A decline in production and activity of intracellular antioxidant enzymes such as superoxide dismutase (SOD) and glutathione peroxidase can also lead to enhanced oxidative stress in DM patients.

Nuclear factor E2-related factor 2 (Nrf2) is a central regulator of intracellular antioxidant response and is a key factor against oxidative stress injury in cardiomyocytes. Nrf2 is widely expressed in the liver, lung, heart, and brain and is considered to be the most important endogenous factor involved in cellular oxidation. Nrf2 translocates to the nucleus and upregulates a series of antioxidant genes, including heme oxygenase-1, SOD, and catalase, thereby reducing cardiac injury [9]. Previous studies have shown significant downregulation of Nrf2 in the left ventricle of the heart of DM patients, and oxidative stress-induced decline in the expression of downstream antioxidant factors heat shock protein 60, heme oxygenase-1, and NADPH in DM myocardial tissue [10,11]. In addition, Nrf2 and its downstream target genes were also downregulated in cardiomyocytes from DM mice (db/db mice), which exacerbated diabetic myocardial dysfunction, myocardial hypertrophy, and inflammatory responses, and also aggravated MIRI [12]. The above evidence suggests that endogenous Nrf2 is not sufficient to resist MIRI. The effect and possible mechanism of enhancing endogenous Nrf2 to reduce myocardial injury in DM can be verified using the Nrf2 agonist dimethyl fumarate (DMF) and the Nrf2 inhibitor ML385 [13] or overexpressing Nrf2.

Nrf2 plays a crucial role in regulating metabolism, mitochondrial function, and oxidative stress [8,14], and oxidative stress significantly affects mitochondrial morphology. Once the dynamic balance of mitochondrial fission/fusion is broken, mitochondria tend to develop toward fission and, consequently, irreversible apoptosis is initiated [15]. Mitochondrial fission mediated by dynamin-related protein 1 (Drp1) plays a critical role in MIRI in non-DM subjects. Also, Drp1-mediated mitochondrial fission was enhanced in the myocardium of DM mice, indicating that Drp1 might be a key molecule associated with MIRI in DM [16].

Therefore, we hypothesized that elevating Nrf2 expression could reduce myocardial susceptibility to I/R injury by improving mitochondrial quality control (mitochondrial fission/fusion). In this study, we established *in vivo* and *in vitro* models of DM-MIRI to investigate whether activation of the Nrf2 signaling pathway could reduce MIRI in DM rats by regulating mitochondrial fission.

## 2 Materials and methods

### 2.1 Experimental animals and grouping

Healthy and clean male SD rats weighing 200–240 g were provided by the Experimental Animal Center of Xinjiang Medical University. Sixty DM rats were randomly divided into 4 groups (15 rats in each group): DM + sham group, DM + MIRI group, DM + MIRI + DMF group, and DM + MIRI + DMF + ML385 group. Seven days before inducing MIRI, DM + MIRI + DMF group received intragastric administration of 25 mg/kg DMF (batch number: C13955800, Merck, Germany) once a day for 7 days before surgery. Thirty minutes before ischemia, DM + MIRI + DMF + ML385 group received intraperitoneal injection of 30 mg/kg ML385 (batch number: M00240, Bio-Leibo Technology Co., Ltd). Fasting blood glucose by tail vein blood sampling and body weight were measured before and after intervention (surgery and drug administration) in each group.


**Ethical Approval:** The use of experimental animals strictly followed the animal protection and use regulations of the center, and was approved by the animal experiment ethics committee of People’s Hospital of Xinjiang Uygur Autonomous Region (KY201803709).

### 2.2 Preparation of a rat model of DM

The DM rat model was constructed as previously reported [17]. Healthy male SD rats were fed with a high-fat diet (60% common forage, 10% lard, 10% sugar, 19% cholesterol, 0.5% edible salt, and 0.5% sesame oil) for 4 weeks. After 12 h of fasting, 30 mg/kg streptozotocin (batch number: 01291613, Sigma, USA) dissolved in citrate buffer at a concentration of 1% (w/v) was injected intraperitoneally at one time, followed by tail vein blood sampling to determine fasting blood glucose. Successful establishment of DM rat model was indicated by fasting blood glucose ≥16.7 mmol/L, and the presence of typical DM symptoms (eating more, drinking more, urinating more often, and weight loss).

### 2.3 Preparation of a rat model of MIRI

DM rats in each group were fasted and not allowed to drink for 12 h. They were anesthetized by intraperitoneally injecting 50 mg/kg of 1% pentobarbital sodium (batch number: WXBB6772V, Sigma, USA). They were then fixed in a supine position for endotracheal intubation, and a small animal ventilator (model: DW2000, Shanghai Jiapeng Technology Co., Ltd, China) for mechanical ventilation and a monitor for continuous monitoring of lead II electrocardiogram (ECG) were connected. The rat was placed in the right lateral decubitus position to allow the chest to be opened at the 4th and 5th intercostal spaces of the left chest. Subsequently, the heart was exposed with a distractor, and the pericardium was incised. After the heartbeat was stable for 15 min, the anterior descending coronary artery of the rat was ligated. The ligation was achieved using a 6-0 suture under a microscope at 2 mm below the lower edge of the left atrial appendage and a latex tube with groove was inserted between the vessel and ligature during ligation. In the rats with successful ischemia, ECG showed ST segment arched elevation or high amplitude of T waves, and the surface of the heart turned pale. After 30 min of ischemia, the ligature was cut and the latex tube was removed to restore coronary blood flow. Successful reperfusion was indicated by the characteristics that the myocardial color at the ischemic site returned to bright red, and the elevated ST segment decreased by more than 50% or the T waves decreased. The reperfusion time was 120 min. By contrast, rats in the DM + sham group were threaded without ligation.

### 2.4 Hematoxylin-eosin (H&E) staining

Myocardial tissues were fixed in 4% paraformaldehyde, dehydrated, embedded in paraffin, and sectioned at 4 µm. Then, conventional H&E staining procedures were performed, and myocardial pathological findings were observed under a light microscope (×200, Carl Zeiss AG, Germany).

### 2.5 Observation of myocardial mitochondrial structure by electron microscopy

Myocardial tissues were cut into small pieces of 0.5 cm^3^ with a blade, and fixed in 3% glutaraldehyde in phosphate buffer (batch number: 10016318, Sinopharm Chemical Reagent Co., Ltd, China). On completion of ultrathin sectioning for electron microscopy, the samples were observed and photographed under a transmission electron microscope (×20,000, Talos F200S, FEI Corporation, USA).

### 2.6 Detection of myocardial injury marker levels

Blood samples (2 mL) were drawn after 120 min of reperfusion. Serum creatine kinase-MB (CK-MB) (E-EL-R1327c, Elabscience Biotechnology Co., Ltd, China), lactate dehydrogenase (LDH) (A020-2, NanJing Jiancheng Bioengineering Institute, China), and cardiac troponin I (cTnI) (E-EL-R1253c, Elabscience Biotechnology Co., Ltd) were detected according to the kit instructions (Elabscience, Wuhan, China).

### 2.7 Detection of oxidative stress marker levels in myocardial tissue

Left ventricular myocardial tissues were collected and snap-frozen in liquid nitrogen and stored in a freezer at −80°C. Malondialdehyde (MDA) content (E-EL-0060c, Elabscience Biotechnology Co., Ltd), ROS content (E-BC-K138-F, Elabscience Biotechnology Co., Ltd), and SOD activity (E-BC-K019-S, Elabscience Biotechnology Co., Ltd) were measured in myocardial tissue homogenate supernatants using the kits. Briefly, MDA concentrations were measured at 450 nm using a microplate reader (Thermo Fisher Scientific, Waltham, USA) according to the manufacturer’s instructions. The ROS content was detected using a fluorescence enzyme marker (Thermo Fisher Scientific) at an excitation wavelength of 500 nm and an emission wavelength of 525 nm. The SOD content was measured at 550 nm using an Ultraviolet visible spectrophotometer (Thermo Fisher Scientific).

### 2.8 RT‐qPCR assay

Myocardial tissue RNA was extracted using an RNA extraction kit (batch number: RP5331, Biofavor Biotechnology Services Co., Ltd, Wuhan, China). The RNA was reverse transcribed to cDNA followed by gene amplification. Reaction conditions were 95°C for 10 min, 95°C for 15 s, and 60°C for 60 s for 45 consecutive cycles. β‐actin was used as an internal reference. The relative expression levels of Nrf2 and Drp1 mRNA were calculated by the 2^−△△CT^ method (Table 1).

**Table 1:** Primer sequences for RT-PCR

Gene	Primer sequences
Nrf2	F 5′-CCATTCCCAGTTACAGT-GTCTT-3′
	R 5′-GATCGATGAGTAAAAATG-GTA-3′
Drp1	F 5′-GAAGTGGTGCAGTG-GAAATGAC-3′
	R 5′-GTTTCTATTGGGAAC-CACTGCC-3′
β-actin	F 5′-GGAGATTACT-GCCCTGGCTCCTA-3′
	R 5′-GACTCATCG-TACTCCTGCTTGCTG-3′

### 2.9 Western blot

Total protein was extracted from left ventricular myocardial tissue or cells by RIPA kit (Batch number: P0010, Biofavor Biotechnology Services Co., Ltd, Wuhan, China), and the concentration of protein was determined by a BCA assay. 40 µg of total protein was electrophoresed, electrotransferred, and then blocked with the blocking solution (TBST containing 5% non-fat dry milk) for 2 h at room temperature on a shaker. Overnight primary antibody incubation with Nrf2 (1:1,000; batch number: 12721, Cell Signaling, USA) and Drp1 (1:1,000; batch number: 12957-1-ap, Proteintech Group, Inc, Wuhan, China) was carried out at 4°C, followed by TBST rinsing (5 times, 5 min/time). Subsequently, corresponding secondary antibodies (1:50,000; batch number: BA1051, Boster Biological Technology Co., Ltd, Wuhan, China) were added for 2 h incubation at 37°C, followed by TBST rinsing (5 times, 5 min/time). The enhanced chemiluminescence enables the development of proteins and the gray values were analyzed with BandScan. The ratio of gray values of target protein bands to β-actin bands was used as the target protein level.

### 2.10 Cell transfection

Nrf2 lentiviral vector (pGLV3-GFP-Nrf2) and its negative control (pGLV3-GFP-NC) were designed and synthesized by Sangon Biological (Shanghai) Co., Ltd. Nrf2 overexpressed H9c2 cells (H9c2-Nrf2) and NC H9c2 cells (H9c2-NC) were also constructed by this company.

### 2.11 High glucose environment and anoxia/reoxygenation treatment for H9c2 cells

H9c2 cells were cultured in high-glucose DMEM medium at a glucose concentration of 30 mmol/L for 12 h. Then, the cells pretreated with high glucose were randomly divided into four groups: H9c2 cells with high glucose treatment (HG-CM group), H9c2 cells with high glucose treatment and anoxia/reoxygenation (A/R) group (A/R group), H9c2-Nrf2 cells with high glucose treatment and A/R (Nrf2 group), and H9c2-NC cells with high glucose treatment and A/R (NC group). The original culture medium in the Petri dish was removed and replaced with sterile PBS buffer. Then, the Petri dish was placed in a closed incubator containing 95% N_2_ and 5% CO_2_ for 3 h. After that, the Petri dish was removed and sterile PBS buffer was discarded. Complete culture medium containing FBS was added, and the samples were placed in a 37°C incubator containing 5% CO_2_ for another 6 h.

### 2.12 CCK-8 assay for cell viability measurement

H9c2 cells in the logarithmic phase were seeded at 5 × 10^3^ cells/mL in 96-well plates and incubated in a 5% CO_2_ incubator at 37°C for 12 h. After treatment and grouping as described above, 10 μL CCK-8 solution was added to each well and incubated for 2 h. The optical density of each well at 450 nm was determined using a microplate reader (Thermo Fisher Scientific, Waltham, USA), and then the cell viability of each group was calculated.

### 2.13 Flow cytometry for apoptosis detection

H9c2 cells were seeded at 1 × 10^6^ cells/mL in 24-well plates. After treatment and grouping as described above, the supernatant was removed, and the cells were collected and resuspended with 100 µL Annexin V-FITC conjugate according to the manufacturer’s instructions. Then, 5 μL Annexin V-FITC staining solution was added and mixed well, and 10 μL propidium iodide staining solution was added. The mixture was shaken slowly and then incubated in the dark at room temperature for 15 min. On completion of the incubation, the samples were immediately detected on a FACSCanto^TM^ II flow cytometer (BD Biosciences). The apoptosis rate was analyzed using FlowJo software.

### 2.14 Detection of oxidative stress marker levels in cell supernatants

Cell supernatants were collected and detected for the contents of MDA (E-EL-0060c, Elabscience Biotechnology Co., Ltd) and SOD (E-BC-K019-S, Elabscience Biotechnology Co., Ltd) using biochemical kits.

### 2.15 Statistical methods

All data were presented as mean value ± standard deviation, and the data were analyzed using GraphPad Prism 8.0.1 software (GraphPad Prism, San Diego, CA). Student’s *t*-test was used for pairwise comparisons and one-way analysis of variance (ANOVA) for comparisons between multiple groups, with *P* < 0.05 indicating a statistically significant difference.

## 3 Results

### 3.1 Changes in body weight and blood glucose before and after intervention

There were no significant differences in body weight and blood glucose between the four groups of DM rats before and after intervention (*P* > 0.05, Table 2).

**Table 2:** Changes in body weight and blood glucose before and after intervention

Groups	Body weight before intervention (g)	Body weight after intervention (g)	Blood glucose before intervention (mmol/L)	Blood glucose after intervention (mmol/L)
DM + sham	336 ± 26	297 ± 22	24.93 ± 0.52	27.24 ± 0.42
DM + MIRI	341 ± 24	300 ± 27	26.62 ± 0.64	28.83 ± 0.23
DM + MIRI + DMF	339 ± 28	306 ± 26	24.82 ± 0.60	29.42 ± 0.43
DM + MIRI + DMF + ML385	347 ± 22	298 ± 23	25.34 ± 0.43	28.64 ± 0.41

### 3.2 DMF reduced myocardial histopathological damage in diabetic rats with MIRI

**Figure 1:**
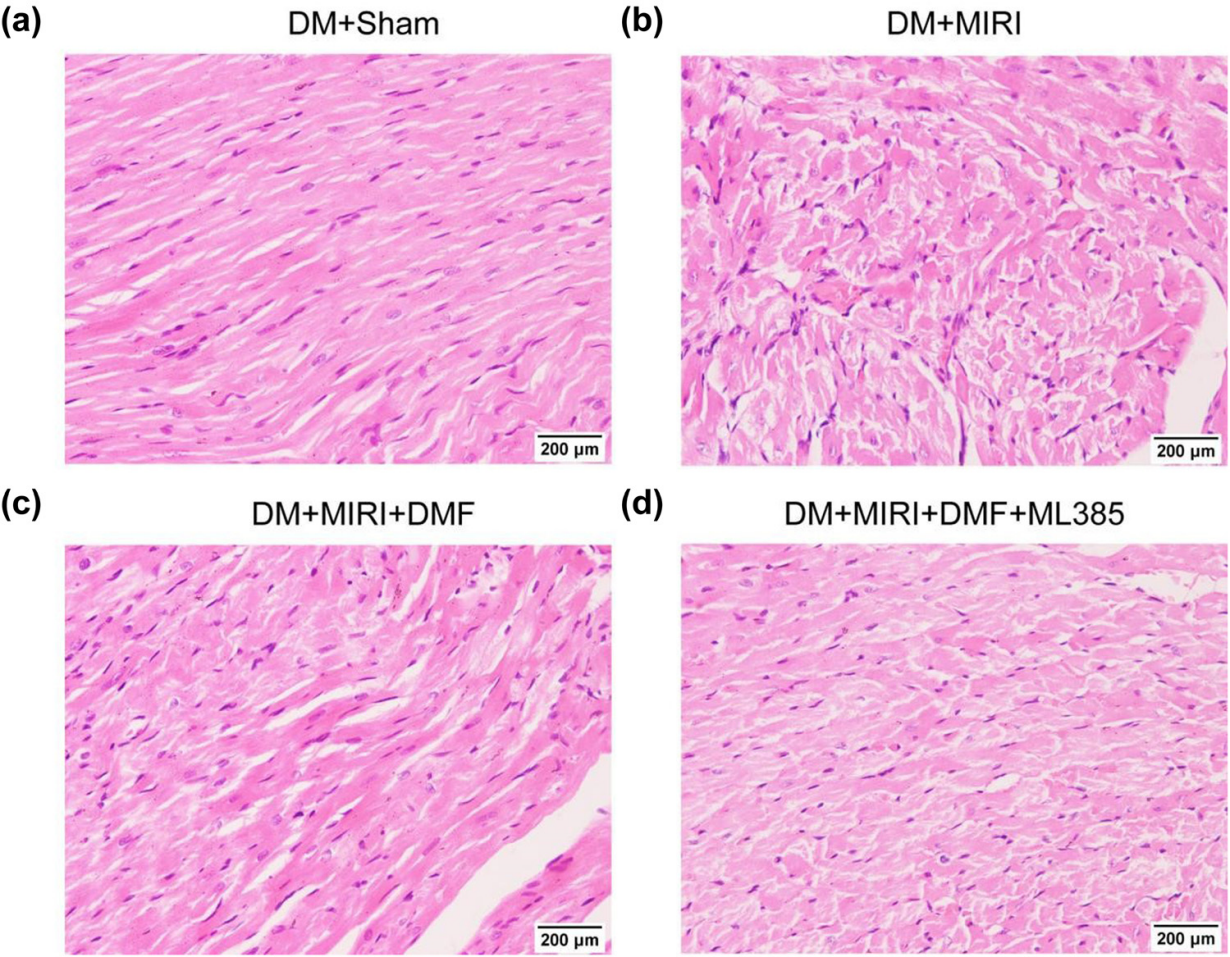
DMF reduced myocardial histopathological damage in diabetic rats with MIRI. (a–d) H&E staining was used to observe the myocardial histopathological damage (×200).

H&E staining showed that the DM + sham group had normal myocardial cell morphology, neatly arranged myocardial fibers, no edema of cardiomyocytes, and no inflammatory cell infiltration. By contrast, the DM + MIRI group and DM + MIRI + DMF + ML385 group had morphological changes in cardiomyocytes, disorganized myocardial fibers and some visible ruptures, significant nuclear pyknosis, and inflammatory cell infiltration. In the myocardial tissue of DM + MIRI + DMF rats, there was a small amount of inflammatory cell infiltration, occasional nuclear pyknosis, mild pathological injury, more complete cell morphological structure, and no lamellar myocardial fiber rupture (Figure 1a–d).

**Figure 2:**
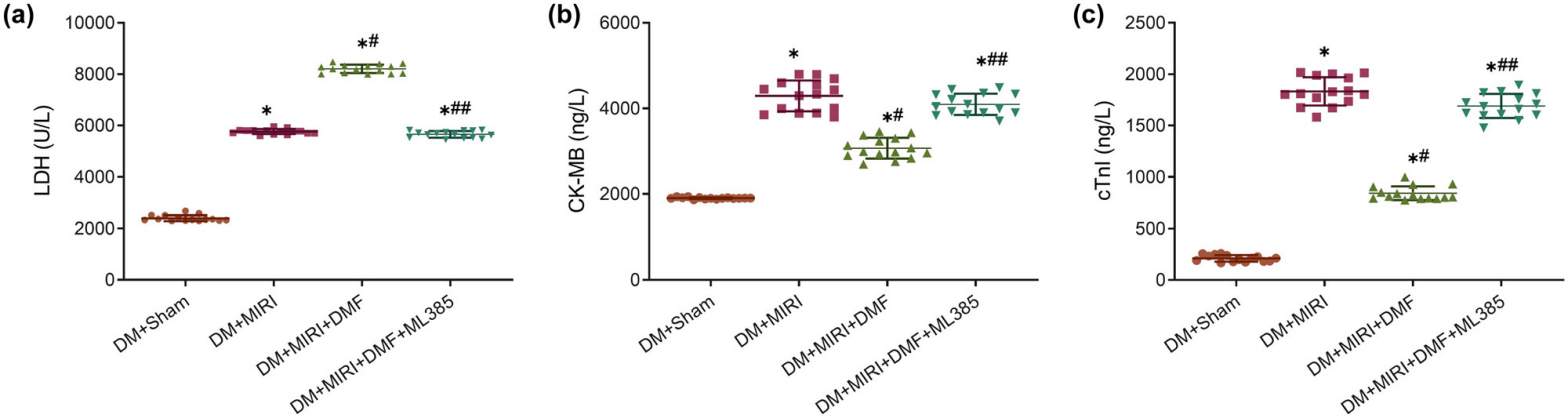
DMF decreased the expression of myocardial injury markers in diabetic rats with MIRI. Serum concentrations of (a) LDH, (b) CK-MB, and (c) cTnI were measured by biochemical kits. *P < 0.05 vs DM + sham group; #*P* < 0.05 and ##*P* < 0.01 vs DM + MIRI group. *n* = 15.

Subsequently, we detected the content of myocardial injury markers in the serum. The results showed that LDH, CK-MB, and cTnI concentrations were increased in the DM + MIRI and DM + MIRI + DMF + ML385 groups compared with DM + sham. DMF treatment could significantly reduce the levels of myocardial injury markers in DM + MIRI rats (*P* < 0.05, Figure 2a–c). These results indicated that treatment with Nrf2 agonist DMF could significantly reduce myocardial injury after myocardial I/R in DM rats.

### 3.3 DMF protected myocardial tissue from oxidative stress injury in diabetic rats with MIRI

**Figure 3:**
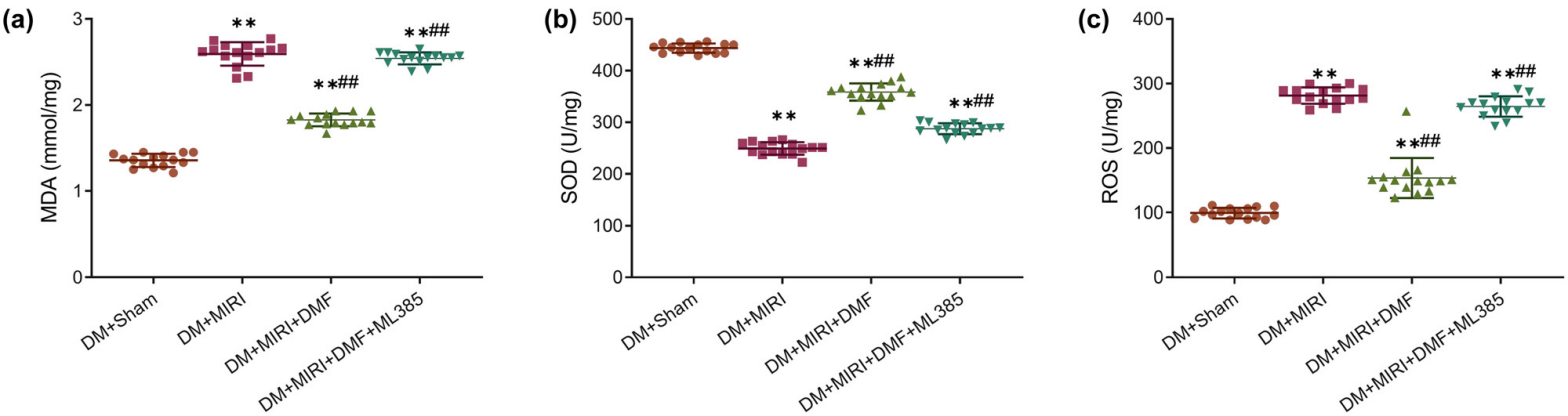
Effect of DMF on changes in the content of oxidative stress markers in myocardial tissue of diabetic rats with MIRI. Biochemical kits were used to detect the levels of (a) MDA, (b) SOD, and (c) ROS in the supernatant of myocardial tissue homogenates. *P < 0.05 vs DM + sham; #*P* < 0.05 and ##*P* < 0.01 vs DM + MIRI. *n* = 15.

As shown in Figure 3a–c, compared with the DM + sham group, MDA and ROS content in the myocardial tissue increased, and SOD activity decreased after MIRI in DM rats (*P* < 0.05). DMF treatment could significantly decrease MDA and ROS contents and increase SOD activity in DM + MIRI rats (*P* < 0.05), indicating that DMF could reduce oxidative stress injury in DM rats with MIRI. However, DMF combined with ML385 treatment significantly inhibited the therapeutic effect of DMF (*P* < 0.05). Collectively, ML385 antagonized the effect of DMF in attenuating oxidative stress following MIRI in DM rats.

### 3.4 Effect of DMF on Nrf2 and Drp1 expression and mitochondrial structure in myocardial tissue of diabetic rats with MIRI

**Figure 4:**
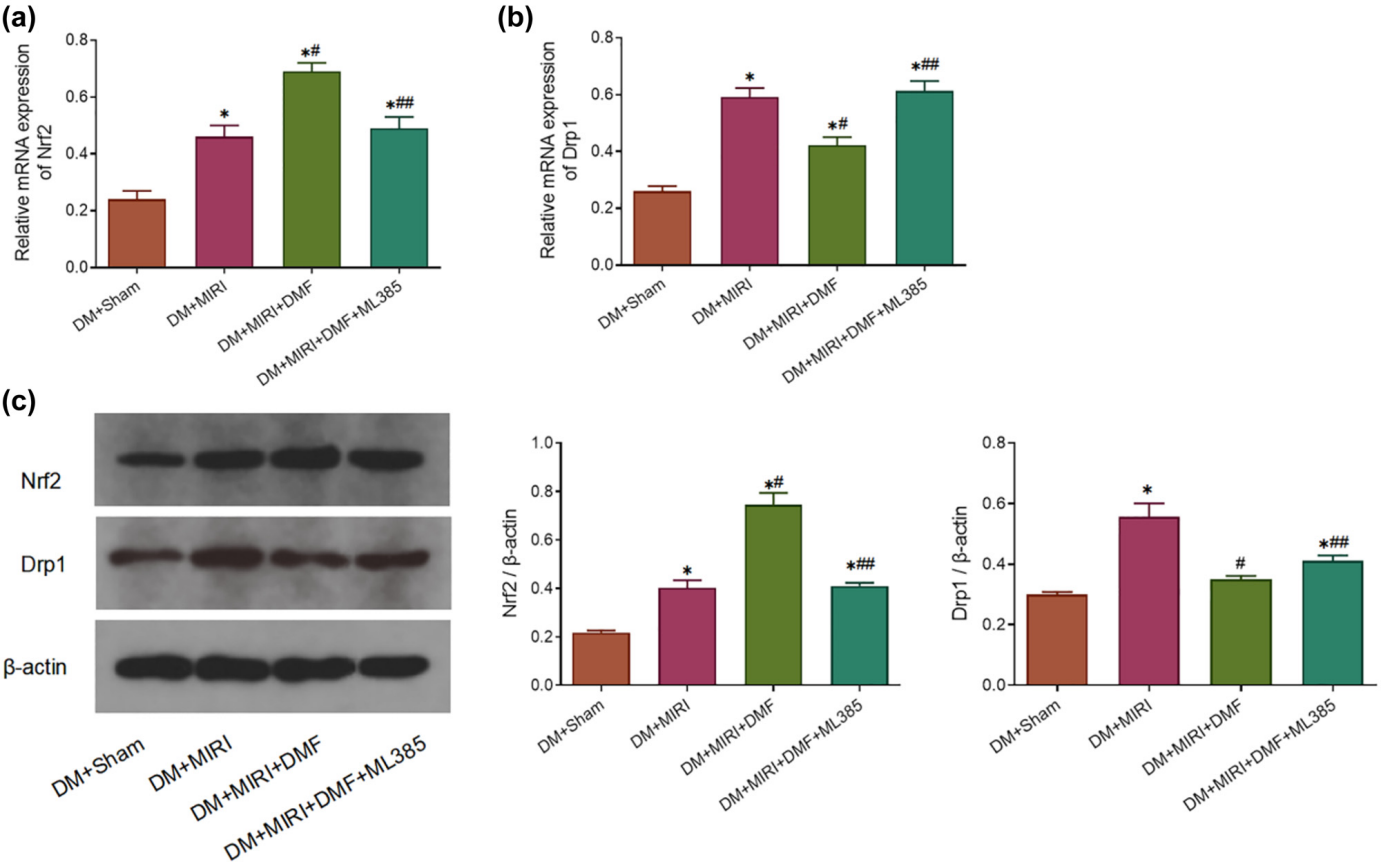
Effect of DMF on Nrf2 and Drp1 expression in myocardial tissue of diabetic rats with MIRI. (a and b) RT-PCR detection of Nrf2 mRNA expression (a) and Drp1 mRNA expression (b) in myocardial tissue; (c) Western blot assay of Nrf2 and Drp1 protein expression. *P < 0.05 vs DM + sham; #*P* < 0.05 and ##*P* < 0.01 vs DM + MIRI. *n* = 15.

**Figure 5:**
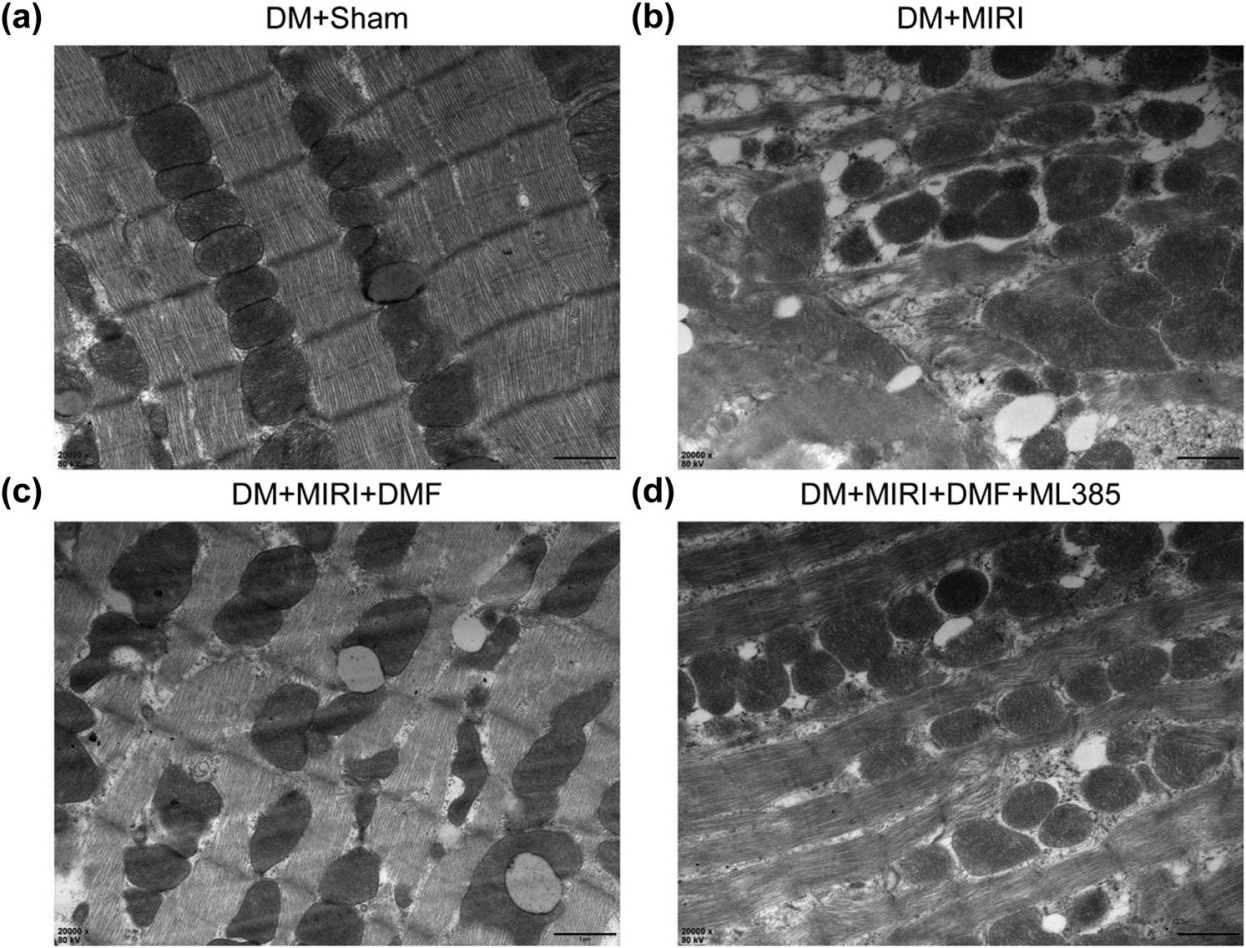
Effect of DMF on mitochondrial structure in myocardial tissue of diabetic rats with MIRI. (a–d) Morphological changes in myocardial mitochondria were observed under transmission electron microscope (×20,000), Scale bar is: 1 μm.

Subsequently, we investigated the expression of Nrf2 and Drp1 in myocardial tissue and myocardial mitochondrial structure after MIRI in DM rats. The results showed that, compared with the DM + sham group, Nrf2 and Drp1 mRNA and protein levels were upregulated (*P* < 0.05) (Figure 4a–c) and myocardial mitochondrial fission was increased (Figure 5a–d) in DM rats after MIRI. DMF treatment significantly increased Nrf2 mRNA and protein expression levels, decreased Drp1 mRNA and protein expression, and reduced myocardial mitochondrial fission in DM + MIRI rats (*P* < 0.05). However, DMF combined with ML385 treatment showed the opposite results to DMF treatment. These results suggested that DMF reduced myocardial injury in DM rats after MIRI by decreasing Drp1 expression and mitochondrial fission in myocardial tissue.

### 3.5 Nrf2 overexpression decreased apoptosis level and oxidative stress damage and increased cell viability in H9c2 cells treated with high glucose and A/R

**Figure 6:**
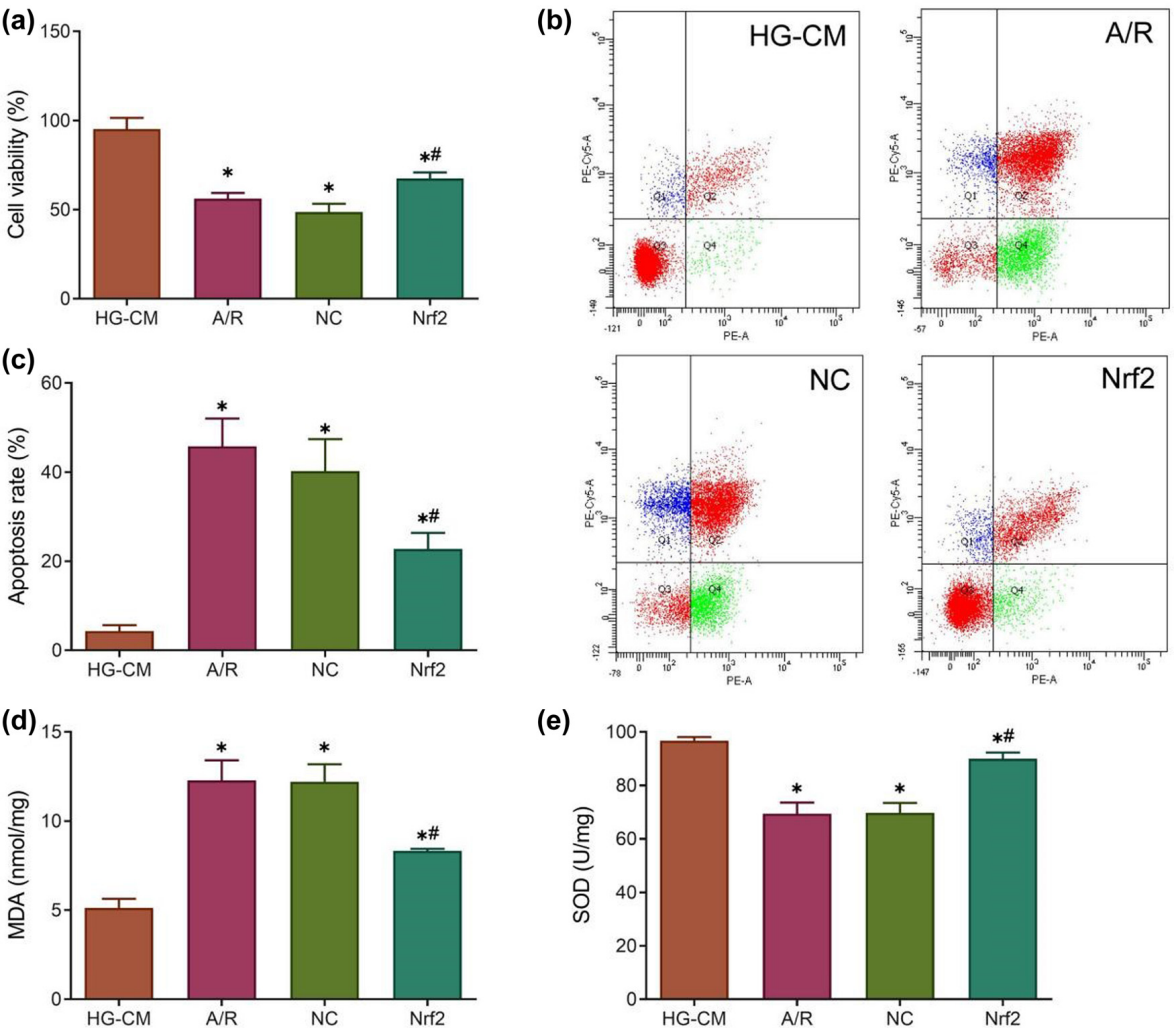
Nrf2 overexpression decreased the apoptosis level and oxidative stress damage and increased cell viability in H9c2 cells treated with high glucose and A/R. (a) CCK-8 detection of cell viability; (b and c) Flow cytometry measurement of cell apoptosis; (d and e) Contents of MDA and SOD in the cell supernatant were detected by biochemical kits. *P < 0.05 vs HG-CM and #*P* < 0.05 vs A/R.

In this study, H9c2 cells treated with high glucose and A/R were used to induce cardiomyocyte injury *in vitro*. Cell viability was measured by CCK-8 assay, and the results showed that H9c2 cell viability was significantly reduced after A/R treatment compared with the HG-CM group (*P* < 0.05), but Nrf2 overexpression significantly antagonized the effect of A/R treatment (*P* < 0.05) (Figure 6a). Further analysis of apoptosis levels revealed that the apoptosis of H9c2 cells increased significantly after A/R treatment compared with the HG-CM group (*P* < 0.05), but Nrf2 overexpression could significantly reduce the apoptotic level of H9c2 cells (*P* < 0.05) (Figure 6b and c).

In addition, we detected SOD and MDA contents in cell supernatants. The results showed a marked rise in MDA content and a significant decline in SOD content after A/R treatment compared with the HG-CM group (*P* < 0.05), and Nrf2 overexpression decreased the level of MDA and increased SOD activity in H9c2 cells compared with the A/R group (*P* < 0.05) (Figure 6d and e). These results indicated that Nrf2 overexpression could reduce the apoptotic level and oxidative stress damage in H9c2 cells treated with high glucose and A/R.

### 3.6 Effect of Nrf2 overexpression on Nrf2 and Drp1 protein expression in H9c2 cells treated with high glucose and A/R

**Figure 7:**
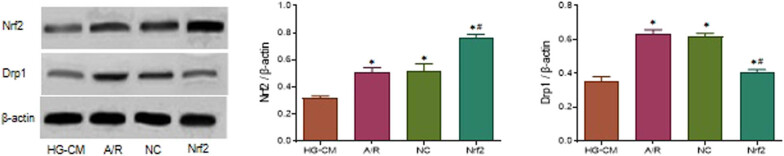
Effect of Nrf2 overexpression on Nrf2 and Drp1 protein expression in H9c2 cells treated with high glucose and A/R. Western blot was used to detect Nrf2 and Drp1 protein expression in cells. *P < 0.05 vs HG-CM and #*P* < 0.05 vs A/R.

Western blot assay results showed that Nrf2 protein expression was significantly increased in cells from the Nrf2 group compared with the NC group, indicating successful transfection of Nrf2. In addition, Nrf2 and Drp1 protein expression was significantly increased in H9c2 cells after A/R treatment compared with the HG-CM group, and Nrf2 overexpression significantly decreased Drp1 protein expression in H9c2 cells compared with the A/R group (*P* < 0.05) (Figure 7). These results suggested that the effects of Nrf2 in H9c2 cells treated with high glucose and A/R might be closely related to inhibition of Drp1 activation.

## 4 Discussion

In the present study, we demonstrated that DM or high glucose significantly increased oxidative stress *in vitro and in vivo*. Drp1-mediated mitochondrial fission and oxidative stress aggravated myocardial injury, while increased Nrf2 expression could antagonize the effects of Drp1.

DM is a metabolic syndrome that seriously endangers human health worldwide, and the World Health Organization predicted that the number of DM patients would increase to 360 million by 2030, accounting for 4.5% of the global population [18]. DM patients have a higher risk of ischemic heart disease and a significantly increased mortality from acute myocardial infarction compared with non-DM patients. Therefore, timely reperfusion therapy plays a crucial role in rescuing dying myocardium, but DM can aggravate MIRI. Such aggravation has been suggested to be associated with mitochondrial dysfunction and oxidative stress [19]. To provide new prevention and treatment strategies, it is necessary to explore the molecular mechanism of MIRI in DM patients. A recent report pointed out that early changes in DM were associated with mitosis, cardiac lipid accumulation, and oxidative stress of mitochondrial origin, especially with altered mitochondrial dynamics [20]. Mitochondrial dynamics are in homeostasis between fission and fusion in the body, and they play a critical role in the pathophysiological mechanism of MIRI [21,22]. Oxidative stress caused by high ROS leads to excessive mitochondrial division [23,24,25], which in turn activates Drp1 and subsequently leads to cardiomyocyte death. Drp1 may be a potential new therapeutic target for cardiac complications in DM cases, but its specific upstream mechanism remains unclear.

Nrf2, a member of the transcription factor family, has a highly conserved basic leucine zipper structure. It is widely expressed in the cardiovascular system and is considered to be the most vital endogenous anti-oxidative stress pathway [26]. Normally, Nrf2 binds to the cytoskeleton-associated protein Keapl present in the cytoplasm. By contrast, in MIRI subjects, a large amount of oxygen-free radical production puts the tissue under oxidative stress, and consequently, Nrf2 dissociates from Keapl and transports from the cytoplasm to the nucleus to regulate the expression of downstream antioxidant factors [27,28]. It has been determined that the regulation of proteasome activity by Keap1-Nrf2 is a new pathway to inhibit mitochondrial fission [15]. These findings suggest that inhibition of mitochondrial fission can restore the balance between mitochondrial fission/fusion and has the potential to be a target for the treatment of diseases associated with mitochondrial dysfunction [29]. In a previous study, we found that the expression of Nrf2 was impaired in DM subjects with MIRI compared with non-DM subjects with MIRI [5]. In this study, we found that when myocardial ischemic injury occurred in DM rats or cells treated with high glucose, upregulation of Nrf2 and Drp1 protein levels, contents of myocardial injury markers (LDH, CK-MB, and cTnI), and contents of oxidative stress products (MDA and ROS) was identified, while the activity of antioxidant SOD was decreased. Additionally, myocardial mitochondrial fission was increased in DM rats after MIRI. Our experimental results confirmed that Nrf2 expression can be activated in case of MIRI; however, endogenous Nrf2 is not sufficient to resist MIRI-induced myocardial injury. DMF, an agonist of Nrf2, is a small molecule with antioxidant, anti-inflammatory, and immunomodulatory effects [30]. Some studies have reported that DMF can activate Nrf2 and upregulate its downstream antioxidant genes to exert myocardial protection [31].

Whether increased Nrf2 expression plays a protective role after MIRI in DM rats was further clarified in this study. Nrf2 expression levels were significantly increased after DMF treatment, and the degree of myocardial pathological damage was alleviated. In addition, DMF treatment downregulated Drp1 expression and the degree of myocardial mitochondrial fission. These results indicate that myocardial protection by DMF is associated with enhanced expression of Nrf2. To determine the relationship of DMF with Nrf2 and Drp1, we preconditioned rats with the Nrf2 inhibitor ML385 intraperitoneally 30 min before ischemia on the basis of the DM + MIRI + DMF group. The combined treatment of DMF and ML385 partially counteracted the myocardial protective effect of DMF alone. Therefore, all these results suggest that activation of Nrf2 is possibly associated with myocardial protection by DMF in DM rats with MIRI, which in turn inhibits Drp1-mediated mitochondrial fission.

Nrf2 plays a crucial role in regulating metabolism, mitochondrial function, and ROS production. Yan et al. [14] have demonstrated that Nrf2 regulated Drp1 stability, thus contributing to the benefits of exercise intervention on age-related skeletal diseases. Sabouny et al. [15] found that increased proteasome activity due to enhanced Nrf2 activity is responsible for Drp1 degradation which occurs in an ubiquitin-independent manner. Hou et al. [32] also showed that mitoquinone, a mitochondria-targeted antioxidant, modulated the Nrf2/Drp1 pathways to attenuate endotoxin-mediated lung injury. In our study, H9c2 cells treated with high glucose and A/R showed a clear increase in apoptotic rate, oxidative stress level, and Drp1 expression. Overexpression of Nrf2 significantly inhibited the effects of high glucose and A/R on H9c2 cells and protected the cells. Thus, Nrf2 has a protective effect on cardiomyocytes by inhibiting Drp1 in DM cases with MIRI.

## 5 Conclusion

In conclusion, DMF plays a protective role after MIRI in DM rats by mediating mitochondrial fission and oxidative stress through activation of Nrf2 and subsequent inhibition of Drp1. Overexpression of Nrf2 similarly achieves protection of H9c2 cells through this mechanism in *in vitro* experiments.
